# *In-situ* growth of superconducting SmO_1−*x*_F_*x*_FeAs thin films by pulsed laser deposition

**DOI:** 10.1038/srep35797

**Published:** 2016-10-21

**Authors:** Silvia Haindl, Kota Hanzawa, Hikaru Sato, Hidenori Hiramatsu, Hideo Hosono

**Affiliations:** 1 Laboratory for Materials and Structures, Institute of Innovative Research, Tokyo Institute of Technology, Mailbox R3-1, 4259 Nagatsuta-cho, Midori-ku, Yokohama, Kanagawa 226-8503, Japan; 2 Physikalisches Institut, Universität Tübingen, 72076 Tübingen, Germany; 3 Materials Research Center for Element Strategy, Tokyo Institute of Technology, Mailbox SE-6, 4259 Nagatsuta-cho, Midori-ku, Yokohama, Kanagawa 226-8503, Japan

## Abstract

Oxypnictide thin film growth by pulsed laser deposition (PLD) is one of many insufficiently resolved issues in the research of iron-based superconductors. Here we report on the successful realization of superconducting SmO_1−*x*_F_*x*_FeAs oxypnictide thin film growth by *in-situ* PLD on CaF_2_ (fluorite) substrates. CaF_2_ acts as fluorine supplier by diffusion and thus enables superconducting oxypnictide thin film growth by PLD. Films are grown heteroepitaxially and characteristically have a broad resistive normal-to-superconducting transition. Best films have onset transition temperatures around 40 K. The proposed *in-situ* PLD film growth offers an alternative and cheap route for the fabrication of iron oxypnictides. PLD becomes now an additional option for iron oxypnictide synthesis.

Discovery of high-temperature superconductivity in iron oxypnictides^1^ that crystallize in the ZrCuSiAs-structure^2^ (so-called 1111) led to surprises, provoked expectations, and keeps on exerting fascination. Their high critical temperatures up to T_*c*_ ≈ 55 K and their high upper critical fields *μ*_0_*H*_*c*2_ > 50 T create an attractive goal for synthesis, engineering and science of oxypnictide thin films, however, their growth has been a challenge ever since 2008^3^. In contrast to the high-temperature superconductor YBa_2_Cu_3_O_7−*δ*_, where film growth by *in-situ* PLD succeeded promptly after its discovery in 1987^4^, *in-situ* PLD of iron oxypnictides has been an unresolved issue and this fact severely impedes the development of iron oxypnictide thin film applications. The furthermore difficult and expensive high-pressure single crystal synthesis of oxypnictides strongly motivates the search for complementary, faster, more feasible and cheaper fabrication techniques^5^.

The main challenge for an *in-situ* PLD growth of superconducting iron oxypnictides is, in first instance, to balance the loss of stoichiometric transfer of high vapor pressure (volatile) elements such as F that is essential for doping and, thus, inevitable for inducing superconductivity in the *parent* compound. In general, PLD is a powerful tool for growing thin films from a stoichiometric source (target)^6^. However, it becomes arbitrarily complicated for volatile species. Off-stoichiometric transfer in PLD^7^ is commonly defeated by an enrichment of the target with the volatile element or by deposition under reactive conditions. For achieving F-doping in the as-grown films we propose here another method where F-doping is obtained via diffusion from the substrate. This approach enables the first successful growth of superconducting iron oxypnictide thin films by *in-situ* PLD.

## From *ex-situ* to *in-situ* PLD

The first film growth attempts of F-doped LaOFeAs by PLD in ultra-high vacuum (UHV) in 2008 succeeded in heteroepitaxy of LaOFeAs on oxide substrates^8^. Unfortunately, the resulting films were undoped and non-superconducting as a result of the volatility of fluorine. Because of the lack of stoichiometric transfer PLD of oxypnictides is sharply constrained. Furthermore, oxypnictide phase formation from the vapor phase is always in contest with the formation of impurity phases such as pure iron, arsenic oxides, iron arsenides, and rare-earth oxides^9^.

An approach based on room-temperature deposition of ROFeAs (R = La, Sm) and a subsequent *ex-situ* heat treatment of the as-grown films in an evacuated silica-glass tube, that contains an additional RO_1−*x*_F_*x*_FeAs pellet, resulted in superconducting films, however with less control over epitaxy^10^,^11^,^12^. Epitaxial RO_1−*x*_F_*x*_FeAs thin films were finally grown by fine tuning the parameters of the heat treatment and due to a rather accidentally formed rare-earth oxyfluoride impurity layer that acts as a seed^13^. The investigation of critical current densities and the upper critical field anisotropy of an epitaxially grown LaO_1−*x*_F_*x*_FeAs thin film with a thickness of 100 nm can thus be viewed as an early milestone^14^. Nevertheless, PLD is used for the creation of nucleation centers on the substrate but the *two-step* route clearly deviates from a typical PLD film growth and suffers from insufficient control of F-doping level^15^.

In the last five years advances in the growth of oxypnictide thin films were made by means of molecular beam epitaxy (MBE) which should be able to master film growth with volatile elements^16^,^17^,^18^. However, also in MBE F-doping has first been introduced via diffusion from a fluorine containing capping layer or from a fluorine containing substrate. Other thin film growth methods such as chemical vapor deposition have not yet found their breakthrough in producing F-doped oxypnictides^19^.

For an advance in PLD of oxypnictide thin films and application-oriented research all above summarized experiences suggest a reactive *all in-situ* PLD process, *i.e.* under tunable fluorine gas supply. This reactive process can only be performed under severe safety regulations, because fluorine gas is highly toxic. In order to tackle the challenge of oxypnictide thin film growth by an *all in-situ* PLD process, we have re-investigated oxypnictide thin film growth on different substrates (and templates) and found, alternatively, a fluorine supplier in CaF_2_ substrates. Our results are directive for future oxypnictide thin film growth efforts.

## Methods

### Thin film deposition

Thin film deposition was carried out in a UHV chamber (base pressure 5 × 10^−9^ mbar) using a Spectra Physics Quanta-Ray INDI pulsed Nd:YAG laser (2*ω*) with a fixed laser repetition rate setting of 10 Hz and a wave-length of 532 nm. Measurements of the pulsed beam energy were performed in front of the entrance window of the UHV chamber before each deposition. Energies of 20–25 mJ used for film deposition result in energy densities on the target surface of approximately 0.9–1.7 Jcm^−2^.

Deposition was performed on different single crystalline oxide substrates, MgO(100) (*a* = 4.21 Å), LaAlO_3_ (100) (*a* = 3.79 Å), and on single crystalline alkaline earth halide CaF_2_ (100) (*a* = 5.46 Å). Furthermore, film growth on Fe and BaFe_2_As_2_ buffered MgO substrates was investigated since the excellent in-plane lattice parameter matching is expected to facilitate epitaxial film growth of the 1111 oxypnictide phase. In the following this excursion on buffer layers is only shortly summarized and our focus will be on the results obtained for F-doped SmOFeAs on CaF_2_ (100) substrates.

Commercially available CaF_2_ substrates (10 × 10 × 0.5 mm^3^) from Furuuchi Chemical Corporation were heated within the vacuum chamber by a high power diode laser system to the deposition temperature and were kept 5 min at this temperature prior to deposition. No special pre-treatment of the substrates was undertaken. The substrate temperature inside the vacuum chamber was monitored by a pyrometer and by a thermocouple. Optimal deposition temperatures are around 860 °C. The target-substrate distance was set to 25–30 mm.

### Target preparation

The polycrystalline SmO_0.9_F_0.1_FeAs target used in the experiments was synthesized by a two-step solid state reaction. All processes except heating were performed in an argon-filled glove box. First, the precursor materials SmAs, Fe_2_As and FeAs were synthesized by mixing elements of Sm, Fe, and As. They were mixed in the chemical composition of Sm:Fe:As = 1:3:3 and heated at 900 °C for 12 h in an evacuated silica tube. The resultant SmFe_3_As_3_ powder was then mixed with dehydrated Sm_2_O_3_ powder, where 10% of Sm_2_O_3_ was replaced by a 1:1 mixture of SmF_3_ and Sm metal for F substitution, following the chemical reaction of SmFe_3_As_3_ + 0.9 Sm_2_O_3_ + 0.1 SmF_3_ + 0.1 Sm → 3 Sm(O_0.9_F_0.1_)FeAs. The mixture compound Sm(O_0.9_F_0.1_)FeAs was pressed and heated in an evacuated silica-tube at 1300 °C for 40 h to obtain a sintered pellet. Phase purity of the resulting PLD target was examined by X-ray diffraction, indicating a small amount of FeAs impurity.

### Thin film characterization

Standard characterization of the grown thin films was carried out by X-ray diffraction on a Rigaku SmartLab and on a Bruker AXS D8 Advance diffractometer, both equipped with Cu K*α* radiation. High resolution X-ray diffractometry (XRD) and reflectivity (XRR) analysis were performed using a Ge 2-bounce monochromator for the incident beam (Rigaku SmartLab). Film thicknesses were determined from Kiessig fringes in XRR. For surface characterization a Bruker AXS MultiMode8 Atomic Force Microscope (AFM) was used in tapping mode with conventional silicon tips on nitride cantilevers (*f*_0_ = 130 ± 30 kHz, *k* = 0.4 Nm^−1^). Images were processed and analyzed with WSxM software^20^.

Surface analysis was also carried out on a JEOL Scanning Electron Microscope (SEM) at working distance of 8 mm and operating with a high voltage of 15 kV.

Electrical resistance measurements were carried out in a Quantum Design Physical Property Measurement System (PPMS) typically in a temperature range between T = 2–300 K and up to applied magnetic fields of *μ*_0_*H* = 9 T. Cu wires of diameter below 100 *μ*m were attached with Ag paste as electrical contacts.

## Results

### Film growth on stable oxide substrates

Iron oxypnictide thin film growth on stable oxide substrates - as already reported in refs 8 and 9 - is characterized by impurity phase formation such as Fe_2_As and Sm_2_O_3_ that strongly compete with the 1111 oxypnictide phase formation. *In-situ* PLD of iron oxypnictides is thus characterized by a constrained window of deposition parameters such as temperature, energy density on the target surface and target-substrate distance. The lack of superconductivity in the films is mainly attributed to the F-loss during deposition but a crucial O- and As-deficiency cannot be excluded.

**Figure 1:**
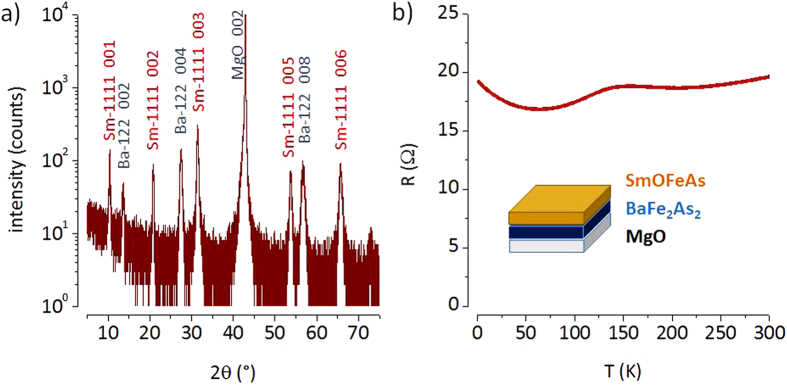
(**a**) High resolution XRD (2*θ*/*ω*-scan) of SmOFeAs/BaFe_2_As_2_/MgO. 00*l* reflections of each phase are indexed. The BaFe_2_As_2_ layer (Ba-122) has a thickness of about 16 nm (*c*_122_ = 12.97 Å). The SmOFeAs layer (Sm-1111) was deposited at 860 °C and is about 36 nm thin (*c*_1111_ = 8.53 Å). (**b**) No superconducting transition is detected in the electrical resistance measured down to 2 K.

Whereas oxypnictide films can be grown epitaxially on BaFe_2_As_2_/MgO templates, films on Fe/MgO always contained a larger fraction of impurity phases. These results contradict the conjecture made by Thersleff that Fe could be a *generic* seed layer for the epitaxial growth of Fe-based superconductors in general^22^. For both templates the absence of a superconducting transition in SmO_1−*x*_F_*x*_ FeAs deposited films can be again explained primarily due to F-losses. Results of the apparently undoped SmOFeAs film are shown in Fig. 1.

### Film growth on CaF_2_

The lack in stoichiometric transfer during PLD for the growth of F-doped iron oxypnictides can be balanced by the use of CaF_2_ substrates that serve as a fluorine supplier during deposition by diffusion. With this working hypothesis superconducting SmO_1−*x*_F_*x*_ FeAs thin films were fabricated on CaF_2_(001) substrates at temperatures around 860 °C. CaF_2_ substrates were used in PLD of iron-based superconducting films with the basic idea of tuning epitaxial strain^23^, but never with the aim of F doping. In contrast, a possible chemical influence of CaF_2_ substrates on the superconducting properties was primarily neglected^3^. Only a few exceptional studies like ref. 24 reported a possible chemical reaction of the CaF_2_ substrate even for iron chalcogenide thin films, where deposition temperatures are typically below 400 °C. Chemical reactions have to be considered at higher deposition temperatures used in our case and in the case of BaFe_2_As_2_ thin film growth.

**Figure 2:**
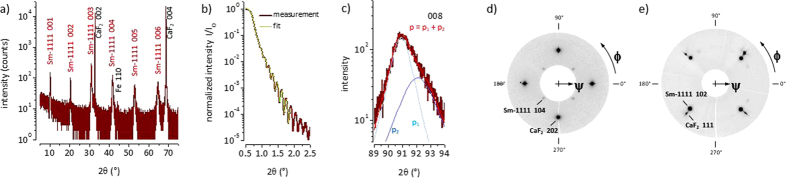
(**a**) High resolution XRD (2*θ*/*ω*-scan) of SmO_1−*x*_F_*x*_ FeAs/CaF_2_. 00*l* reflections of the 1111 phase and the substrate are indexed. The obtained lattice parameter is *c*_1111_ = 8.66 Å. Small amount of Fe impurity is found as indicated by the Fe 110 reflection. (**b**) A total film thickness of 58.4 nm was evaluated from a fit of the normalized XRR intensity in the range of 2*θ* = 0.6°–1.8°. (**c**) 008 Bragg reflection with total maximum at 2*θ* ≈ 90.95° as a superposition of two extremal profiles p_1_ (with maximum at 2*θ* ≈ 90.9°) and p_2_ (with maximum at 2*θ* ≈ 92.1°). (**d**) Pole figure (*ψ, ϕ*) for 2*θ* = 47.0° ± 1.0° with CaF_2_ 202 and SmO_1−*x*_F_*x*_FeAs 104 reflections. (**e**) Pole figure (*ψ, ϕ*) for 2*θ* = 30.0° ± 1.2° with CaF_2_ 111 and SmO_1−*x*_F_*x*_FeAs 102 reflections.

In the following we demonstrate that thin film growth of superconducting iron oxypnictides is realized on CaF_2_. No impurity phases except a small amount of Fe were detected by XRD (Fig. 2a). The best film has an onset transition temperature, *T*_*c*,*on*_ near 40 K. The *c*-axis lattice parameters of the PLD grown films (8.63–8.66 Å) are comparable to the reported *c*-axis lattice parameters for MBE grown films (8.55–8.65 Å)^18^ but generally larger than 8.495 Å (*x* = 0), 8.428 Å (*x* = 0.1)^25^, or 8.488–8.498 Å (*x* = 0.2)^26^, in sintered polycrystalline powder samples and also larger than the *c*-axis in a single crystal (8.468 Å for *x* = 0.14)^5^.

Asymmetric Bragg reflections indicate the presence of diffusion layers or gradients. The lattice parameter change with film thickness can be estimated by a fit of the reflection intensity composed of a superposition of several peak profiles. The asymmetry intensifies with higher order, exemplarily the 008 reflection is shown (Fig. 2c). According to a simple fit the estimated relative change in the *c*-axis lattice parameter is slightly less than 1%.

Heteroepitaxial growth of SmO_1−*x*_F_*x*_FeAs on CaF_2_ was confirmed by pole figure measurements (Fig. 2d,e) and is characterized by the following orientation relationship: (001)[100]SmO_1−*x*_F_*x*_FeAs||(001)[110]CaF_2_. SmO_1−*x*_F_*x*_FeAs grows with the *c*-axis perpendicular to the substrate surface and its basal plane *a* × *a* is rotated by 45° in-plane versus the unit cell of CaF_2_.

**Figure 3:**
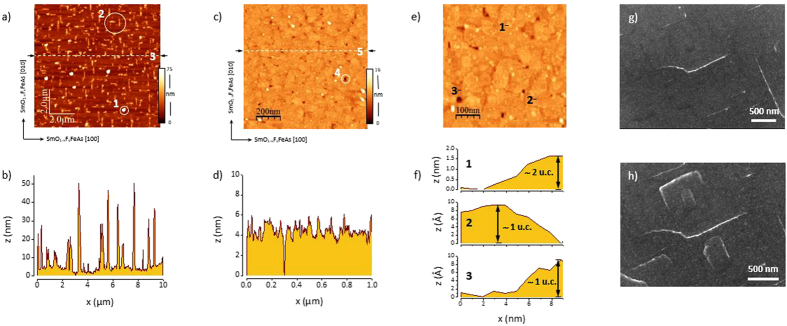
(**a**) AFM image (10 × 10 *μ*m^2^) of the film surface (film deposition time = 10 min) scanned with a rate of 0.5 Hz and 512 samples/line along the crystallographic [100] direction of the SmO_1−*x*_F_*x*_FeAs film. Apart from (1) droplets the film surface topography is characterized by (2) a line-shaped defect structure oriented along the crystallographic [100] and [010] direction of SmO_1−*x*_F_*x*_FeAs. The root-mean-squared roughness (rms) is 8.5 nm. (**b**) Profile of a vertical line scan (3) across the defect structure. The estimated size of the defects is about 500 × 150 × 50 nm^3^. (**c**) AFM image (1 × 1 *μ*m^2^) of a thinner film (deposition time = 5 min) scanned with a rate of 1.5 Hz and 512 samples/line along the crystallographic [100] direction of the SmO_1−*x*_F_*x*_FeAs film. Line-shaped defects do not appear here but holes (4) are still present in the microstructure after island coalescence. The rms roughness of the film surface within this scan is 0.8 nm. (**d**) Profile of a vertical line scan (5) across the defect structure. (**e**) AFM image with scan area of 500 × 500 nm^2^ (compare Fig. 3c). The rms roughness is 0.74 nm. (**f**) Selected profiles of terraces with step sizes of approximately 1 and 2 unit cells. (**g**) SEM image of a film surface with initial cracks and (**h**) induced cracks during scanning of the electron beam on the same magnified area of the film. The cracks (white contrast) appear quickly within several seconds.

Apart from droplets, *i.e.* particles of larger size typically observed on the surface of PLD grown films, the global surface topography is characterized by defects that evolve along the crystallographic [100] and [010] directions of the SmO_1−*x*_F_*x*_FeAs film (Fig. 3a–d). These line-shaped defects appear in films grown on CaF_2_ substrates and originate very likely from cracks. Such cracks are also observed to appear during scanning electron microscopy (SEM) where whole crack networks are induced under the scanning electron beam obviously by local heat generation in the CaF_2_ substrate. SEM characterization of the films is thus a destructive procedure. Two consecutive scans demonstrate the appearance of defects within a short time (Fig. 3g,h). It is therefore plausible that the observed defect structure in the surface topography is caused by the bursting of the film surface. These defects increase the surface roughness (global rms ≈ 10 nm in a 10 × 10 *μ*m^2^ scan including droplets vs. local rms ≈ 1 nm in a in a 1 × 1 *μ*m^2^ scan), and also deteriorate the current flow (and critical currents) in the films leading most likely to current percolation.

The growth mode distinguishes from a layer-by-layer and resembles an island (Volmer-Weber) growth, where terrace-like structures with step sizes of approximately 1 and 2 unit cells can be identified (Fig. 3e,f).

Because of low film growth rates (≤ 1 Å s^−1^) deposition times of 5–10 min are required for film thicknesses of 20–50 nm. With increasing deposition time (>5 min) at elevated temperatures crack formation in the as-grown films is observed by AFM imaging that might be a result of outgassing fluorine leading to a bursting of the film layer along its crystallographic *a*-axis. CaF_2_ substrates are not yet perfectly suitable for technological applications.

**Figure 4:**
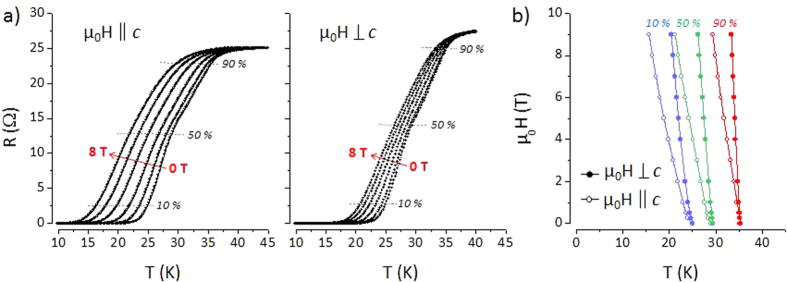
(**a**) Resistive transitions R(T) for zero field and magnetic fields (0.5, 2, 4, 6, 8 T) applied parallel to the *c*-axis and perpendicular to the *c*-axis. The criteria of 90%, 50% and 10% of normal resistance above the transition are indicated by dashed lines. (**b**) Magnetic phase diagram *μ*_0_*H*_*c*2_(T) evaluated for different criteria.

Electrical resistance of the films measured in van-der-Pauw geometry for applied magnetic fields parallel and perpendicular to the film *c*-axis (Fig. 4a). Qualitatively, the resistive transitions are very broad. The usually applied resistance criteria for 90%, 50%, and 10% of the normal resistance result in T_*c*,90_ ≈ 35 K, T_*c*,50_ ≈ 28 K, and T_*c*,10_ ≈ 24 K. The broad transitions and the changes in the slope of R(T) within the normal-to-superconducting transition are characteristic for an inhomogeneous film due to F-doping gradients. Current percolation caused by the observed defects can have additional effects on broadening. The upper critical field (Fig. 4b) extracted from data of the 10% criterion has moderate slopes of dH_*c*2_/d*T* ≈ −2.7 TK^−1^ (−1.3 TK^−1^) for *μ*_0_*H*_*c*2_||*ab (μ*_0_*H*_*c*2_||*c*). The maximum slope of d*H*_*c*2_/d*T* ≈ −5.5 TK^−1^ estimated from the 90% criterion for *μ*_0_*H*_*c*2_||*ab* corresponds to reported values in sintered powders^22^.

## Discussion and Conclusion

The experiments have confirmed that superconductivity in iron oxypnictides depends sensitively on the stoichiometry of the rare earth oxide layer in the 1111 unit-cell. O-deficiency turns out to be an additional drawback for the *in-situ* PLD process of oxypnictides. Successful growth of superconducting iron oxypnictides by PLD must, therefore, supply enough oxygen and fluorine during film growth. These results are in accordance with investigations of O-deficient oxypnictides, RO_1−*δ*_FeAs, that are not superconducting^21^. A precise stoichiometric control stays still a technological challenge for these materials and might be realizable only after optimization or in a reactive fabrication process. The essential supply of fluorine during film growth for doping and stabilizing the rare earth oxide layers is enabled by a diffusion process. This new solution deviates from the usual PLD philosophy that relies on the stoichiometric transfer of material from the target to the substrate. In using F-diffusion provoked by the substrate, latter becomes an additional source of material in the described *in-situ* PLD process.

The choice of brittle CaF_2_ substrates is not yet optimal for applications of Fe-based superconductor thin films, a fact that has been widely neglected in the thin film growth of Fe-based superconductors in general. In future CaF_2_ should be replaced by other materials that act as F-suppliers and, simultaneously, have better mechanical properties. As a remark: The observed enhancement in critical temperatures in Co-doped BaFe_2_As_2_ thin films grown on CaF_2_ is very likely a result of a fluorine interdiffusion from the substrate to the film followed by a change in stoichiometry and lattice parameters. Similar defects (cracks) as described above appear in the surface morphology of Ba(Fe_1−*x*_Co_*x*_)_2_As_2_ films, too. Considering the difficult and rare synthesis of iron oxypnictide single crystals or thin films in general the importance of having a standard fabrication process for oxypnictides is evident. Our results indicate once more the requirement of a highly advanced process for machining iron oxypnictides into competitive superconducting applications. The above presented iron oxypnictide film fabrication route by *in-situ* PLD allows film growth and further development. With an *in-situ* PLD process for SmO_1−*x*_F_*x*_FeAs the already developed concepts of coated conductor technology can be adapted as it was demonstrated by a first *proof-of-concept* for FeSe_1−*x*_Te_*x*_^27^ and Co-doped BaFe_2_As_2_^28^ superconductors already. Since film growth by MBE is still expensive and difficult to upscale towards an industrial level, a suitable PLD fabrication process represents a substantial step towards a reliable application-oriented research on iron-oxypnictides.

To summarize, the here reported successful *in-situ* growth of superconducting oxypnictide thin films by PLD on CaF_2_ substrates represents a significant progress in the fabrication of F-doped oxypnictide thin films. *In-situ* PLD of superconducting iron oxypnictides seemed to be impossible due to a lack in stoichiometric transfer and the loss of fluorine as a dopant. Fluorine supply by diffusion from the CaF_2_ substrate is, however, able to introduce sufficient doping. We have demonstrated that the use of the substrate as additional material source represents a methodological advance in PLD against material loss due to volatile components. Furthermore, the diffusion process offers at present a cheap solution to the problem of F-doping during film growth and circumvents chemical hazards of a fully reactive process.

## Additional Information

**How to cite this article**: Haindl, S. *et al. In-situ* growth of superconducting SmO_1−*x*_F*_x_*FeAs thin films by pulsed laser deposition. *Sci. Rep.* **6**, 35797; doi: 10.1038/srep35797 (2016).
